# Protection Induced in Broiler Chickens following Drinking-Water Delivery of Live Infectious Laryngotracheitis Vaccines against Subsequent Challenge with Recombinant Field Virus

**DOI:** 10.1371/journal.pone.0137719

**Published:** 2015-09-14

**Authors:** Mesula G. Korsa, Glenn F. Browning, Mauricio J. C. Coppo, Alistair R. Legione, James R. Gilkerson, Amir H. Noormohammadi, Paola K. Vaz, Sang-Won Lee, Joanne M. Devlin, Carol A. Hartley

**Affiliations:** 1 The Asia-Pacific Centre for Animal Health, Faculty of Veterinary and Agricultural Sciences, The University of Melbourne, Parkville, Victoria, Australia; 2 College of Veterinary Medicine, Konkuk University, Seoul, Republic of Korea; University of Maryland, UNITED STATES

## Abstract

Infectious laryngotracheitis virus (ILTV) causes acute upper respiratory tract disease in chickens. Attenuated live ILTV vaccines are often used to help control disease, but these vaccines have well documented limitations, including retention of residual virulence, incomplete protection, transmission of vaccine virus to unvaccinated birds and reversion to high levels of virulence following bird-to-bird passage. Recently, two novel ILTV field strains (class 8 and 9 ILTV viruses) emerged in Australia due to natural recombination between two genotypically distinct commercial ILTV vaccines. These recombinant field strains became dominant field strains in important poultry producing areas. In Victoria, Australia, the recombinant class 9 virus largely displaced the previously predominant class 2 ILTV strain. The ability of ILTV vaccines to protect against challenge with the novel class 9 ILTV strain has not been studied. Here, the protection induced by direct (drinking-water) and indirect (contact) exposure to four different ILTV vaccines against challenge with class 9 ILTV in commercial broilers was studied. The vaccines significantly reduced, but did not prevent, challenge virus replication in vaccinated chickens. Only one vaccine significantly reduced the severity of tracheal pathology after direct drinking-water vaccination. The results indicate that the current vaccines can be used to help control class 9 ILTV, but also indicate that these vaccines have limitations that should be considered when designing and implementing disease control programs.

## Introduction

Infectious laryngotracheitis (ILT) is a contagious upper respiratory tract disease of chickens that causes significant economic losses in poultry industries around the world [1–3]. The disease is caused by an alphaherpesvirus, infectious laryngotracheitis virus (ILTV), which is classified taxonomically as *Gallidherpesvirus 1* [4]. In some outbreaks mortality rates of up to 70% have been reported [5]. Attenuated ILTV vaccines have been widely used to control the disease. However, these vaccines have several limitations, including insufficient attenuation [6], transmission of vaccine virus to unvaccinated birds [7,8], increased virulence after bird-to-bird transmission [9] and also incomplete protection in vaccinated birds [10,11].

Recently, two genetically distinct field strains (class 8 and 9 ILTV viruses) were detected in Australia using PCR-RFLP genotyping [12]. Evidence from whole genome sequence analysis of the three vaccine strains in use in Australia, along with the genome of these newly emerged strains, confirmed that the class 8 and 9 strains emerged as a result of natural (field) recombination between the recently introduced European-origin vaccine strain (Serva ILTV, MSD Animal Health) and the original Australian vaccine strains (SA-2 and A20 ILTV, Zoeitis) [13]. The novel recombinant class 9 ILTV strain became the predominant field strain in important poultry producing regions in Victoria, Australia, largely displacing the previously dominant class 2 ILTVs [12] and continues to cause significant outbreaks of disease in commercial poultry flocks [13]. Recent studies have shown that, compared to class 2 ILTV, class 9 ILTV has enhanced replication kinetics, increased virulence and enhanced potential for horizontal transmission. These differences may help to explain the dominance of class 9 ILTV in the field [14].

Another factor that could contribute to the dominance of class 9 ILTV in the field is the extent to which the virus can be controlled using vaccination. The ability of vaccines to protect birds against challenge with class 9 ILTV has not been investigated previously. This study aimed to examine the extent to which four different live attenuated ILTV vaccines could protect commercial broiler birds against challenge with virulent class 9 ILTV. In order to remain relevant to field situations, this study aimed to use conditions similar to those that occur in the field, where possible.

## Materials and Methods

### Experimental design and virus strains used in this study

Approval for this study (Animal Ethics ID 1312956.1) was granted by the Animal Ethics Committee of the Faculty of Veterinary and Agricultural Sciences, The University of Melbourne. One hundred and twenty 10-day-old broilers (obtained from a commercial supplier at 1 day of age) were individually identified with numbered wing-tags and weighed on the day of vaccination (10 days old). Six groups of 20 birds each were placed in separate isolator units and were provided with feed and water *ad libitum*. Three groups were vaccinated with either Serva ILTV, SA-2 ILTV or A20 ILTV via drinking water according to manufacturers' instructions. A fourth group was similarly vaccinated with a glycoprotein G deleted candidate vaccine (ΔgG ILTV) [15] via drinking water at a dose of 1.0 x 10^5^ plaque forming units (PFU)/bird. The remaining two groups (negative and positive control groups) were mock vaccinated by addition of sterile cell culture medium to their drinking water. Immediately after vaccination, after the drinkers containing the vaccine had been removed from the isolators, five age-matched unvaccinated birds were added to each group. Twenty days after vaccination, all birds (including the contact-exposed birds) were inoculated with 1.0 x 10^3^ PFU of virulent recombinant class 9 ILTV, except for the birds in the negative control group, which were mock-challenged with sterile cell culture medium. For challenge, half of the virus dose was inoculated into the trachea and half of the dose was administered via eye-drop. The class 9 strain of ILTV had been propagated and titrated as described previously [16].

Four days after challenge, tracheal and conjunctival swabs were collected from all birds to assess viral replication. In addition, five birds that had been vaccinated directly in each group were selected at random and euthanised by exposure to an overdose of an inhalant anesthetic agent (halothane). These birds were weighed and proximal tracheal sections were collected and processed for histopathological examination as described previously [17]. Seven days after challenge all remaining birds were euthanized, weighed and samples collected as described above. The severity of virus-induced tracheal lesions, virus detection and replication in the trachea and conjunctiva, and body weight changes were used to assess protection.

### Tracheal histopathology

Transverse sections of proximal trachea were collected, processed and stained with haematoxylin and eosin as described previously [14]. The severity of the histopathological lesions were scored from 0 (absent) to 4 (severe) as described previously [18] by two operators blinded to the group of origin of each of the sections.

### Virus detection and quantification

DNA was extracted from tracheal and conjunctival swabs using the QIAxtractor Vx virus kit (Qiagen) and a QIAextractor automated system (Qiagen) as described previously [14]. Positive and negative controls were included on each extraction plate. Infectious laryngotracheitis DNA was detected and quantified in the extracted DNA using real-time quantitative PCR and primers that amplify 113 bp of the UL15 gene of ILTV, as described previously [19]. A 10-fold dilution series of the UL15 sequence cloned into pGEM-T (Promega) was included in duplicate on each plate to enable estimation of the ILTV genome concentration in each of the extracted samples, with the lower limit of detection for the assay defined as 52 genome copies per reaction. Viral genome concentrations were log_10_ transformed for statistical analysis.

### Statistical analysis

Minitab 17 (Minitab Inc, 2010), GraphPad Prism 6 (GraphPad Prism Software) and Excel 2007 (Microsoft) were used to analyse data. Mann-Whitney tests were performed to compare the lesion scores determined by histopathological examination. One-way analyses of variance, in conjunction with Dunnett’s Multiple Comparisons tests, were used to compare the viral genome concentrations and percentage body weight gains for the different groups. The normality assumption was assessed using normal probability plots, and equality of variance was checked using Levene’s test. Fisher’s exact test was used to compare the proportions of ILTV positive birds in each groups. A two-tailed p ≤ 0.05 was considered to be significant.

## Results

### Protection in broilers directly vaccinated via drinking water

Results from assays for virus detection and virus quantification, and tracheal histopathological examination in directly vaccinated birds, four days after challenge, are summarized in Table 1. No significant differences in the severity of tracheal histopathological lesions were detected between groups. Viral genome concentrations were significantly lower in the tracheas of birds vaccinated with Serva, A20, SA-2 or ΔgG ILTV than is the unvaccinated-challenged (positive control) group. In contrast, viral genome concentrations in the conjunctiva did not differ significantly between any of the challenged groups. The proportion of birds in which virus was detected in the conjunctiva and trachea varied between groups. Within the vaccinated groups, the lowest proportions of ILTV positive birds were seen in the group vaccinated with A20 ILTV (for detection of virus in the trachea) and in the group vaccinated with SA-2 ILTV (for detection of virus in the conjunctiva).

**Table 1:** Results from assays for virus detection and virus quantification, and tracheal histopathological examination in birds directly vaccinated with different ILTV vaccines, four days after challenge .

Group		Median tracheal histopathology score (range)	Mean log_10_ viral genome copies/reaction ± S.D.^¶^		Proportion of birds positive for ILTV	
Vaccine	Challenge		Trachea	Conjunctiva	Trachea	Conjunctiva
None	None	1 (1–2) ^a^	1.7 ± 0 ^a^	1.7 ± 0 ^a^	0/17 ^a^	0/17 ^a^
None	Class 9	2 (1–3) ^a^	3.4 ± 2.2 ^b^	1.8 ± 0.4 ^a^	9/20 ^b^ ^,^ ^c^	2/20 ^a^ ^,^ ^c^
Serva	Class 9	2 (1–3) ^a^	3 ± 1.8 ^a^	1.9 ± 0.4^a^	9/19 ^b^	7/19 ^b^ ^,^ ^c^
A20	Class 9	2 (1–3) ^a^	1.8 ± 0.3 ^a^	1.8 ± 0.2 ^a^	3/20 ^a^ ^,^ ^c^	5/20 ^b^ ^,^ ^c^
SA-2	Class 9	2 (0–3) ^a^	2.6 ± 0.9^a^	1.7 ± 0 ^a^	12/18 ^b^	0/18 ^a^
ΔgG	Class 9	2 (1–5) ^a^	2.9 ± 2.0 ^a^	1.8 ± 0.4 ^a^	6/19 ^b^ ^,^ ^c^	1/19 ^a^

^a,b,c^ Values marked with the same superscripts in the same column were not significantly different (p > 0.05).

^¶^ S.D = standard deviation. The lower limit of detection of the assay used to detect and quantify viral genome was 52, or 10^1.72^, genome copies per reaction.

Results from assays for virus detection and virus quantification, and tracheal histopathological examination in directly vaccinated birds, seven days after challenge, are summarized in Table 2. Birds that received the SA-2 ILTV vaccine had significantly less severe upper tracheal histopathology than birds in all other challenged groups. Viral genome concentrations were significantly lower in the tracheas of birds vaccinated with Serva, A20, SA-2 or ΔgG ILTV, than in birds in the unvaccinated-challenged (positive control) group. Viral genome concentrations were significantly lower in the conjunctivas of the birds vaccinated with Serva, A20 or SA-2 ILTV, than in those of the birds in the unvaccinated-challenged (positive control group). The proportion of birds in which virus was detected in the conjunctiva and trachea varied between groups. Within the vaccinated groups, the lowest proportions of ILTV positive birds were seen in the groups vaccinated with Serva or A20 ILTV (for detection of virus in the trachea) and in the group vaccinated with SA-2 ILTV (for detection of virus in the conjunctiva).

**Table 2:** Results from assays for virus detection and virus quantification, and tracheal histopathological examination in birds directly vaccinated with different ILTV vaccines, seven days after challenge.

Group		Median tracheal histopathology score (range)	Mean log_10_ viral genome copies/reaction ± S.D^¶^		Proportion of birds positive for ILTV	
Vaccine	Challenge		Trachea	Conjunctiva	Trachea	Conjunctiva
None	None	2 (0–2) ^a^	1.7 ± 0 ^a^	1.7 ± 0 ^a^	0/12 ^a^	0/12 ^a^
None	Class 9	3 (0–5) ^b^	4.6 ± 1.9^b^	2.9 ± 1.3 ^b^	13/14 ^b^	12/14 ^b^
Serva	Class 9	3 (2–5) ^b^	2.1 ± 0.9 ^a^	1.9 ± 0.5 ^a^	4/14 ^a^ ^,^ ^c^	3/14 ^a^ ^,^ ^c^
A20	Class 9	3 (1–5) ^b^	2.1 ± 0.8 ^a^	1.9 ± 0.4 ^a^	4/15 ^a^ ^,^ ^c^	3/15 ^a^ ^,^ ^c^
SA-2	Class 9	1 (0–4) ^a^	2.8 ± 1.3 ^a^	1.7 ± 0 ^a^	8/13 ^b^ ^,^ ^c^	0/13 ^a^
ΔgG	Class 9	3 (0–5) ^b^	2.8 ± 1.6 ^a^	2.8 ± 1.6 ^b^	6/15 ^c^	8/15 ^c^ ^,^ ^b^

^a,b,c^ Values marked with the same superscripts in the same column were not significantly different (p > 0.05).

^¶^ S.D = standard deviation. The lower limit of detection of the assay used to detect and quantify viral genome concentrations was 52, or 10^1.72^, genome copies per reaction.

Percentage weight gains in directly vaccinated birds at three different time points (20 days after vaccination, four days after challenge and seven days after challenge) are summarized in Table 3. No significant differences in percentage weight gain were detected between the groups at any of these time points.

**Table 3:** Percentage body weight changes between the day of vaccination and 20 days after vaccination, and between the day of challenge and days four and seven after challenge, in birds directly vaccinated with different ILTV vaccines.

Group		Mean percentage body weight change ± S.D^¶^					
Vaccine	Challenge	N	20 dpv	N	4 dpc	N	7 dpc
None	None	17	381 ± 64 ^a^	5	27 ± 6 ^a^	12	54 ± 8 ^a^
None	Class 9	20	404 ± 91 ^a^	5	15 ± 8 ^a^	14	48 ± 15 ^a^
Serva	Class 9	19	372 ± 98 ^a^	5	33 ± 6 ^a^	14	56 ± 8 ^a^
A20	Class 9	20	414 ± 88 ^a^	5	29 ± 12 ^a^	15	50 ± 6^a^
SA-2	Class 9	18	429 ± 84 ^a^	5	30 ± 3 ^a^	13	48 ± 14 ^a^
ΔgG	Class 9	20	380 ± 80 ^a^	5	28 ± 14 ^a^	15	51 ± 17 ^a^

^a^ Values marked with the same superscripts in the same column were not significantly different (p > 0.05).

^¶^ SD = standard deviation, dpv = days post vaccination, dpc = days post challenge, N = number of birds.

### Protection in broilers that were contact-exposed to vaccinated birds

The results from assays for virus detection and quantification four days after challenge in birds that were contact-exposed to vaccinated chickens, or contact-exposed to mock-vaccinated chickens in the negative and positive control groups, are summarized in Table 4. No significant differences in viral genome concentration, or in the proportion of ILTV positive birds, were seen between groups.

**Table 4:** The results from assays for viral detection and quantification four days after challenge in birds that were contact-exposed to vaccinated chickens.

Group		Mean log_10_ viral genome copies/reaction ± S.D ^¶^		Proportion of birds positive for ILTV	
Vaccine	Challenge	Trachea	Conjunctiva	Trachea	Conjunctiva
None	None	1.7 ± 0 ^a^	1.7 ± 0 ^a^	0/5 ^a^	0/5 ^a^
None	Class 9	3.3 ± 2.1 ^a^	2.0 ± 0.6 ^a^	3/5 ^a^	1/5 ^a^
Serva	Class 9	1.7 ± 0 ^a^	1.7 ± 0.1 ^a^	0/4 ^a^	1/4 ^a^
A20	Class 9	1.7 ± 0 ^a^	1.7 ± 0 ^a^	0/5 ^a^	0/5^a^
SA-2	Class 9	1.9 ± 0.4^a^	1.7 ± 0 ^a^	1/4 ^a^	0/4 ^a^
ΔgG	Class 9	4.0 ± 2.7 ^a^	1.7 ± 0 ^a^	2/4 ^a^	0/4 ^a^

^a^ Values marked with the same superscripts in the same column were not significantly different (p > 0.05).

^¶^ S.D = standard deviation. The lower limit of detection of the assay used to detect and quantify viral genome was 52, or 10^1.72^, genome copies per reaction.

Results from assays for virus detection and virus quantification, and tracheal histopathological examination seven days after challenge in birds that were contact-exposed to vaccinated birds are summarized in Table 5. At this time after challenge, birds that were contact-exposed to chickens vaccinated with A20 or ΔgG ILTV had significantly less severe tracheal histopathology scores than birds that were contact exposed to mock vaccinated chickens in the positive control group. Furthermore, birds that were contact exposed to chickens vaccinated with Serva, A20 or SA-2 ILTV had significantly lower ILTV genome concentrations in the trachea than birds that were contact exposed to mock vaccinated chickens in the positive control group. In contrast, no significant reduction in ILTV genome concentrations were seen in the conjunctiva of birds that were contact exposed to vaccinated chickens in any of the vaccinated groups, compared to birds that were contact exposed to mock-vaccinated chickens in the positive control group. The proportions of contact-exposed birds in which virus was detected in the conjunctiva and trachea varied between groups. Within the vaccinated groups, the lowest proportion of ILTV positive contact-exposed birds was in the SA-2 ILTV vaccinated group for detection of virus in the trachea, and in the A20, Serva and ΔgG ILTV vaccinated groups for detection of virus in the conjunctiva.

**Table 5:** Results from assays for virus detection and virus quantification, and tracheal histopathological examination seven days after challenge in birds that were contact-exposed to vaccinated chickens.

Group		Median tracheal histopathology score (range)	Mean log_10_ viral genome copies/reaction ± S.D^¶^		Proportions of birds positive for ILTV	
Vaccine	Challenge		Trachea	Conjunctiva	Trachea	Conjunctiva
None	None	1 (0–1) ^a^	1.7 ± 0 ^a^	1.7 ± 0 ^a^	0/5 ^a^	0/5 ^a^
None	Class 9	4 (2–4) ^b^	4.7 ± 0.9 ^b^	3.1 ± 1.4 ^b^	5/5 ^b^	5/5 ^b^
serva	Class 9	2 (2–4) ^b^ ^,^ ^c^	1.9 ± 0.4 ^a^	2.3 ± 1.1 ^a^ ^,^ ^b^	1/4 ^a^	1/4 ^a^
A20	Class 9	2 (1–4) ^c^	2.0 ± 0.6 ^a^	2.1 ± 0.8 ^a^ ^,^ ^b^	1/5 ^a^	1/5 ^a^
SA-2	Class 9	3.5 (1–5) ^b^ ^,^ ^c^	1.7 ± 0 ^a^	2.9 ± 0.8 ^b^	0/4^a^	3/4 ^b^
ΔgG	Class 9	2.5 (1–4) ^c^	4.7 ± 2 ^b^	1.9 ± 0.3 ^a^ ^,^ ^b^	3/4 ^b^	1/4^a^

^a,b,c^ Values marked with the same superscripts in the same column were not significantly different (p > 0.05).

^¶^ S.D = standard deviation. The lower limit of detection of the assay used to detect and quantify viral genome was 52, or 10^1.72^, genome copies per reaction.

Percentage weight gains in birds contact-exposed to vaccinated chickens, at two different time points (20 days after vaccination and seven days after challenge) are summarized in Table 6. The only significant difference was in the group that were contact exposed to birds that received the A20 ILTV vaccine. This group had a significantly lower weight gain 20 days after vaccination compared with all other groups.

**Table 6:** Percentage body weight changes between the day of vaccination and 20 days after vaccination, and between the day of challenge and day seven after challenge, in birds that were contact exposed to vaccinated chickens.

Group		Mean percentage body weight change ± S.D ^¶^			
Vaccine	Challenge	N	20 dpv	N	7 dpc
None	None	5	350 ± 37^a^	5	53 ± 9 ^a^
None	Class 9	5	329 ± 42^a^	5	49 ± 14 ^a^
Serva	Class 9	4	358 ± 57^a^	4	61 ± 10 ^a^
A20	Class 9	5	309 ± 67^b^	5	51 ± 9 ^a^
SA-2	Class 9	4	393 ± 64^a^	4	47 ± 4 ^a^
ΔgGV	Class 9	4	401 ± 142^a^	4	36 ± 16 ^a^

^a,b,^ Values marked with the same superscript in the same column at a given time point were not significantly different (p > 0.05).

^¶^ S.D = standard deviation, dpv = days post vaccination, dpc = days post challenge, N = number of birds.

## Discussion

This study aimed to evaluate the ability of attenuated ILTV vaccines to control recombinant, class 9 ILTV under conditions similar to those that occur in the field. Importantly we applied the vaccine via drinking water. Drinking water vaccination is a preferred method for mass delivery of vaccine to large broiler flocks due to the ease and cost effectiveness of application. However, under field conditions, some birds may not consume drinking water containing the vaccine, or may receive a suboptimal dose of the vaccine because of reduced consumption. These birds, therefore, remain either unvaccinated or incompletely vaccinated [10,11,20]. Other factors may also limit the effectiveness of vaccines delivered via drinking water in poultry, including equipment limitations, poor water quality and sub-optimal preparation and handling of the vaccine [21]. The birds that are not fully vaccinated by drinking water may become infected with vaccine virus following horizontal transmission from vaccinated birds [22], or may remain naïve, creating a small susceptible population within the flock. In order to simulate the lack of uniform vaccination that can occur in commercial flocks vaccinated by drinking water, the present study included five unvaccinated, age-matched birds that were placed in-contact with vaccinated birds in each of the groups immediately after vaccination. Their subsequent protection against challenge with virulent virus was assessed. This challenge virus was administered via intra-tracheal inoculation and via eye-drop in order to simulate the respiratory and ocular routes of infection that occur under field conditions [5].

The study used commercial broiler chickens, instead of specific-pathogen free (layer-type) chickens, in order to ensure its relevance to field situations and commercial broiler industries. Previous studies have demonstrated immunological differences between broiler and layer types of chickens. Broilers produce a strong short-term humoral response, whereas layer-type chickens produce a long-term humoral response in conjunction with a stronger cellular response. These features in broilers appear to be a consequence of genetic selection for economically important traits [23]. In this study birds were vaccinated at 10 days of age, consistent with common field vaccination practices in Australia, and challenged at 30 days of age, an age at which ILT outbreaks have commonly been seen in broilers in Australia [22]. Age at vaccination and age at challenge are important parameters that can influence protection and disease expression. Previous studies have shown poorer immune responses in chickens vaccinated before two weeks of age due to the immaturity of their cell-mediated immunity, rather than effects of maternally derived antibodies, which are not protective against ILTV [24,25]. However the short growth cycle in commercial broiler production systems often requires vaccinations to be performed at a younger age than would be immunologically ideal, and so we used a younger age of vaccination in this study. As antibodies are not protective against ILTV this study did not assess serum antibody levels in the vaccinated chickens.

The results from our study showed that, under conditions resembling field conditions, all the attenuated vaccines induced a level of protection against challenge with recombinant class 9 ILTV in chickens directly vaccinated via drinking water. All vaccines reduced the amount of detectable virus in the trachea at both four and seven days after challenge compared to unvaccinated birds. All vaccines also reduced the proportion of birds that were positive for the presence of ILTV DNA in conjunctival and/or tracheal swabs seven days after challenge. Furthermore the SA-2, A20 and Serva ILTV vaccines reduced the amount of virus detected in the conjunctiva seven days after challenge. Reducing the level of virus replication within flocks is important for the control of ILT, as this is likely to reduce the viral load in the environment and potentially decrease the risk of spread beyond the infected flock to new flocks or farms. However it is important to note that this study only assessed viral load using qPCR detection of viral DNA, which is unable to discriminate between viable and unviable virus [17]. Detection of viable virus would require virus isolation in either cell culture or embryonated eggs, however this can be impractical in large studies such as this current study. Also the qPCR assay did not discriminate between DNA from the challenge virus and DNA from vaccine viruses. It is possible that some DNA from the vaccine viruses may have been detectable after challenge. The future development of new diagnostic tools to differentiate and quantitate vaccine and challenge viruses present in mixed infections would be beneficial for investigating this further.

Interestingly, only SA-2 ILTV reduced the severity of tracheal lesions following challenge. Although the SA-2 ILTV strain is known to be highly immunogenic, it is also less attenuated than some other ILTV vaccines, so it is not normally recommended for use in broilers because of concerns about its safety in these birds [15,17,26]. No significant vaccine safety concerns were noted in this study following SA-2 inoculation, however in the field factors such as stocking rates, housing conditions, and concurrent infection with other pathogens may influence disease expression.

In contact-exposed birds, all groups of birds that were contact-exposed to vaccinated chickens showed some degree of protection against challenge compared to birds that were grouped with unvaccinated chickens. This contrasts with a previous study from the USA [27] in which none of the contact-exposed birds were protected against challenge. The differing outcomes of our study and this previous study could be due to differences in the challenge and vaccine strains used between studies and also differences in experimental design, particularly the ratio of in-contact birds to directly vaccinated birds (1:1 in the previous study, 1:4 in this study) that may have created conditions more favourable for transmission of vaccine virus from directly vaccinted to contact-exposed in our study. In our study, this protection was only seen seven days after challenge and included reduced levels of tracheal pathology (in birds contact-exposed to A20 or ΔgG ILTV vaccinated chickens), reduced concentrations of detectable viral DNA in the trachea (in birds contact-exposed to Serva, A20 or SA-2 ILTV vaccinated chickens) or reduced proportions of birds that had detectable ILTV DNA in conjunctival and/or tracheal swabs (in birds contact-exposed to Serva, A20, SA-2 or ΔgG ILTV vaccinated chickens). This protection was presumably due to horizontal transmission of vaccine virus from directly-vaccinated birds to in-contact birds, although this transmission was not directly assessed. Although transmission of vaccine virus to naïve birds can have potential benefits in terms of inducing a level of protection against challenge, there is also potential for some vaccine strains of virus to revert to higher levels of virulence following bird-to-bird passage [8,9] or the potential for their involvement in recombination events with other strains of ILTV [13]. For these reasons field vaccination programs strive to achieve optimal, direct vaccination of all birds to generate uniform protection.

Unexpectedly, no significant difference was seen in percentage weight gain between groups directly vaccinated with the different vaccines in this study. This is in contrast to results from a previous study of class 9 ILTV in which unvaccinated-challenged birds had significantly decreased weight gain, compared to negative control birds at 6 days after challenge [14]. This could be explained by the different environmental conditions that were required for this present study. In particular, light intensity and duration were restricted in order to facilitate the control of aggressive (pecking) behavior seen in some of the chickens. This pecking behavior also necessitated euthanasia (using halothane) of some birds in order to ameliorate suffering, in accordance with animal ethics approval for this work. This resulted in different numbers of birds per group at some time points. In broiler chickens, light restriction programs have been shown to decrease weight gain [28] and so it is possible that these measures to control pecking also restricted weight gain. This may have prevented differences in weight gain between groups being expressed.

In the unvaccinated-challenged birds the assay for virus detection and quantification, and tracheal histopathological examination, revealed some apparent differences in viral replication and disease progression associated with class 9 ILTV compared to another strain of ILTV (CSW-1 ILTV) assessed in previous studies. Following inoculation of class 9 ILTV, the highest viral genome concentrations were seen in the trachea seven days after challenge. The proportions of ILTV positive birds were also highest seven days after challenge and tracheal lesions were most severe seven days after challenge. In studies utilizing the CSW-1 strain of ILTV (a virulent field virus commonly used as an experimental challenge strain in Australia), tracheal pathology and virus replication reach their highest levels four days after challenge, with most virus cleared from the tracheal mucosa by seven days after challenge [15]. The results from this study, in conjunction with previously reported results showing the presence of tracheal pathology and virus replication up to 21 days after infection with class 9 ILTV [14], suggest that the duration of infection and disease is extended for class 9 ILTV. This could be linked to the dominance of class 9 ILTV in the field.

Taken together the results from this study indicate that the currently available attenuated vaccines and the glycoprotein G deleted candidate vaccine can be used to help control class 9 ILTV when delivered by drinking water. However it is important to note that neither the protection induced in the directly-vaccinated birds, nor that induced in birds that were contact-exposed to vaccinated birds, was complete. This may help to explain the persistence of class 9 ILTV infection and disease in commercial poultry flocks of Australia, despite the widespread use of vaccination programs similar to those employed in this study. The results highlight the wisdom of combining vaccination programs with other disease control measures, such as biosecurity measures, in order to improve ILT control. Our study also highlights the need to continue to seek improvements in ILTV vaccines and vaccine delivery methods in order to achieve improved protection against challenge with virulent virus.
